# Micellization of Photo-Responsive Block Copolymers

**DOI:** 10.3390/polym9090396

**Published:** 2017-08-26

**Authors:** Oliver Grimm, Felix Wendler, Felix H. Schacher

**Affiliations:** 1 Institute of Organic Chemistry and Macromolecular Chemistry (IOMC), Friedrich-Schiller-University Jena, Humboldtstraße 10, D-07743 Jena, Germany; oliver.grimm@uni-jena.de (O.G.); felix.wendler@uni-jena.de (F.W.); 2 Jena Center for Soft Matter (JCSM), Friedrich-Schiller-University Jena, Philosophenweg 7, D-07743 Jena, Germany

**Keywords:** block copolymers, photo-responsive, self-assembly, micellization

## Abstract

This review focuses on block copolymers featuring different photo-responsive building blocks and self-assembly of such materials in different selective solvents. We have subdivided the specific examples we selected: (1) according to the wavelength at which the irradiation has to be carried out to achieve photo-response; and (2) according to whether irradiation with light of a suitable wavelength leads to reversible or irreversible changes in material properties (e.g., solubility, charge, or polarity). Exemplarily, an irreversible change could be the photo-cleavage of a nitrobenzyl, pyrenyl or coumarinyl ester, whereas the photo-mediated transition between spiropyran and merocyanin form as well as the isomerization of azobenzenes would represent reversible response to light. The examples presented cover applications including drug delivery (controllable release rates), controlled aggregation/disaggregation, sensing, and the preparation of photochromic hybrid materials.

## 1. Introduction and Scope

Stimuli-responsive materials are capable of undergoing (reversible) changes with regard to physico-chemical properties such as solubility, polarity, charge, charge density, size or shape. Often, this is exploited in applications, where either response is translated into a certain action or where changes in environmental conditions are reported as or transformed into an optical or chemical readout. Examples are diagnostics, drug delivery, tissue engineering, or even triboelectric materials [1]. Among the different stimuli available, light is attracting more and more attention as it can be controlled both spatially and temporally with utmost precision. Moreover, careful adjustment of the used wavelength allows precisely controlling the amount of energy introduced into the system that is exposed to light. This can be crucial to allow certain processes to occur or to avoid irreversible photo damage to the material. The latter case has to be especially considered if biomedical applications are in focus and tissue integrity or cell viability has to be maintained. Here, the Near infra-red (NIR) window (λ = 650–900 nm) is highly attractive as it allows penetration depths of up to 10 mm through human skin and subcutaneous regions. Absorption by blood constituents is reduced and tissue damage or phototoxic effects are minimized [2,3].

Whereas examples for polymeric materials that respond to changes in pH (e.g., polyelectrolytes) [4] or temperature (e.g., poly(*N*-isopropylacrylamide)) [5] are manifold, fewer reports deal with photo-responsive polymers and block copolymers. Nevertheless, the last decade has shown that more and more research groups are working on strategies to implement such properties using different approaches; for a general overview, we direct the reader to recent review articles by Zhao, Gohy and Bertrand [6,7,8].

This review focuses on photo-responsive block copolymers and nanostructured materials generated thereof via self-assembly in selective solvents, whereby most examples report on processes in aqueous media using amphiphilic block copolymers. For bulk applications, the reader is referred to a recent review by Gohy and Bertrand dealing with photo-responsive surfaces, thin films, and gels/hydrogels [8] and an earlier book chapter discussing specifically azo-functionalized block copolymers in the solid state [9]. During this review, we have subdivided the specific examples: (1) according to the wavelength at which the irradiation has to be carried out; and (2) according to whether irradiation with light of a suitable wavelength leads to reversible or irreversible changes in material properties. Thereby, an irreversible change could be the photo-cleavage of a nitrobenzyl ester [10], whereas the photo-mediated transition between spiropyran and merocyanin [11] form would represent reversible response to light. The examples presented cover applications including drug delivery (controllable release rates), controlled aggregation, sensing, and the preparation of photochromic hybrid materials.

## 2. Block Copolymers in General

Block copolymers represent a class of materials where at least two different monomers are arranged in a sequential manner. As a result, one single material can combine the properties of all involved monomer units. One further peculiarity of block copolymers is that, contrary to statistic, random, or gradient copolymers, the intrinsic immiscibility of unlike polymeric segments leads to microphase separation and thereby a straightforward access to nanostructured materials. This holds true for different (micro) environments, i.e., in the bulk or in solution [12,13].

The synthesis and the even today steadily increasing availability of block copolymers originates in the discovery of living polymerization techniques by Michael Szwarc, more specifically the possibility to produce segmented polymeric materials in a sequential manner and, even more important, without unwanted termination or transfer reactions during the polymerization [14,15]. Whereas ionic (anionic, cationic, and anionic/cationic ring-opening polymerization) techniques are still rather demanding in terms of monomer choice, purification procedures, or the tolerance towards functional groups, another huge step forward in the variety of block copolymer compositions and functionalities occurred after the development of reversible deactivation radical polymerization (RDRP) techniques. Here, the most prominent examples are atom transfer radical polymerization (ATRP) [16,17,18], nitroxide mediated polymerization (NMP) [19], and reversible addition fragmentation chain transfer polymerization (RAFT) [20,21]. With constant evolution of these techniques, monomers or monomer combinations previously not being accessible could be realized, and a large variety of functional groups were incorporated into polymeric materials and block copolymers of different architecture. Today, polymer and material chemists can rely on a versatile toolbox of reliable polymerization techniques to prepare materials of defined composition, controllable architecture, and narrow dispersity.

Initially, block copolymers were regarded as rather exotic materials, mainly due to (still) increasing synthetic demand, but, during the last 20 years, more and more research groups developed interest in block copolymers. This can be mainly assigned to the straightforward control over both domain size and morphology of block copolymer-based nanostructured materials in the bulk and in solution. Both can be precisely adjusted by molecular parameters such as molar mass, volume fraction of the respective segments, overall architecture, and also segment rigidity (this also includes the presence of crystallizable blocks). Additional elements of control include strategies to influence the self-assembly process itself (“pathway”) [22], or to exploit suitable chemical tools to specifically address individual domains or even the block junction [23,24] between two adjacent segments in block copolymer nanostructures [25,26].

## 3. λ-Dependent Photo-Response in Block Copolymer Nanostructures in Solution

**Figure 1:**
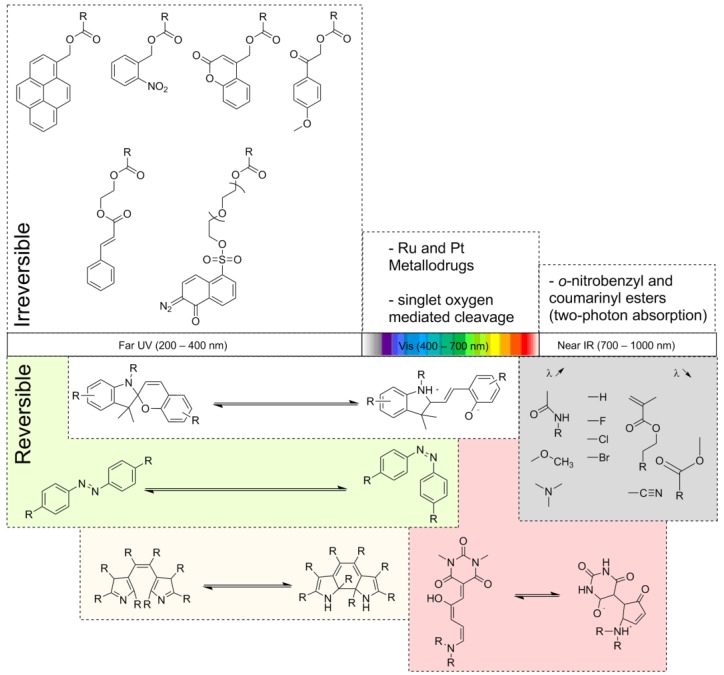
Schematic representation of relevant chromophores that have been incorporated into block copolymers; we distinguish between reversible/irreversible photo-response as well as different excitation regimes: Far UV (200–400 nm), Vis (400–700 nm) and Near IR (700–1000 nm); the grey part depicts different functional groups that are capable of influencing the wavelength at which chromophores respond.

In the following sections, we try to give an overview over relevant photo-responsive chromophores that have been incorporated into block copolymers. We decided to distinguish between examples that refer to reversible or irreversible response upon irradiation with light, as well as with reference to other review articles in this field [6,7,8]. Furthermore, we have grouped the respective articles according to the wavelength at which the irradiation has to be carried out. Thereby, three wavelength regimes have been defined, Far UV (200–400 nm), Vis (400–700 nm) and Near IR (700–1000 nm), and the most relevant chromophores are depicted in Figure 1.

### 3.1. Far UV (200–400 nm)

#### 3.1.1. Reversible

In general, UV light within the range of 200–400 nm provides enough energy to break bonds, induce cis–trans isomerizations, and enable cycloaddition reactions. The most important molecule classes discussed within this section as chromophores are azobenzenes [27,28], spiropyrans [29], cinnamoyl esters [30], and diarylethenes [31].

**Figure 2:**
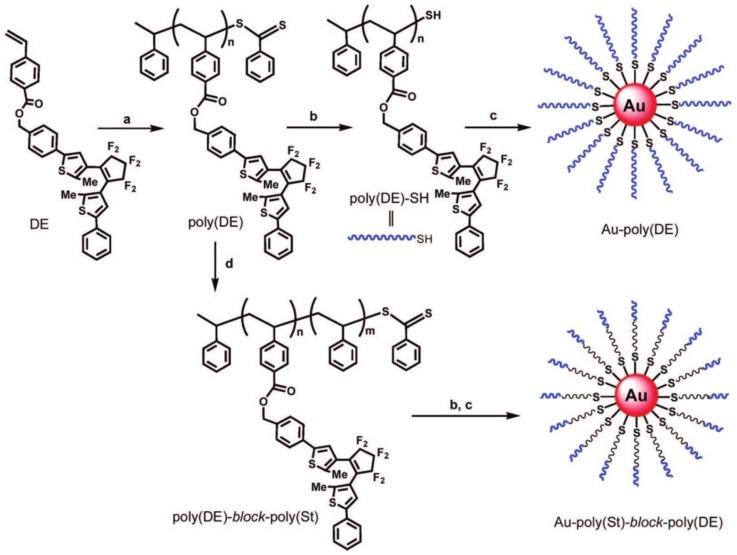
Synthetic scheme for the preparation of Au-poly(diarylethene) and Au-polystyrene-*block*-poly(diarylethene) hybrid nanoparticles. Reprinted with Permission from Ref. [37]. Copyright 2008 American Chemical Society.

The photochromism of heterocyclic 1,2-diarylethenes as a subclass of fulgides has been known since 1905 [32], and the substitution pattern of these photochromic systems was considerably extended in 1988 by Irie and Mohri [33]. The ring closure of single molecule diarylethenes is typically triggered at 325 nm (the back reaction to the cis-form often occurs upon irradiation at 488 nm) [34], but depending on the substitution pattern this wavelength can vary from 313 to 405 nm (correspondingly, the back reaction is triggered from 405 to 546 nm) [33,35]. These photo-responsive diarylethenes can be incorporated into polymers either in the main chain or in the side chain. Some of them could be polymerized, as shown by Stellacci et al. [36] and a redshift of the absorption spectra was observed if compared to the monomer. The modification of a suitable diarylethene with a polymerizable styrene group enabled Nishi et al. to synthesize poly(diarylethene)-*block*-polystyrene diblock copolymers via reversible addition fragmentation chain transfer polymerization (RAFT) [37]. The RAFT endgroup was cleaved off and the thiol was attached to a gold nanoparticle to form photochromic hybrid nanoparticles in toluene (Figure 2). The same technique and also photo-responsive monomer was used by Kobatake [38] to prepare poly(diarylethene)-*block*-PNIPAAm using surface-initiated polymerization from silica nanoparticles. These nanoparticles were investigated both in water and in THF.

**Figure 3:**
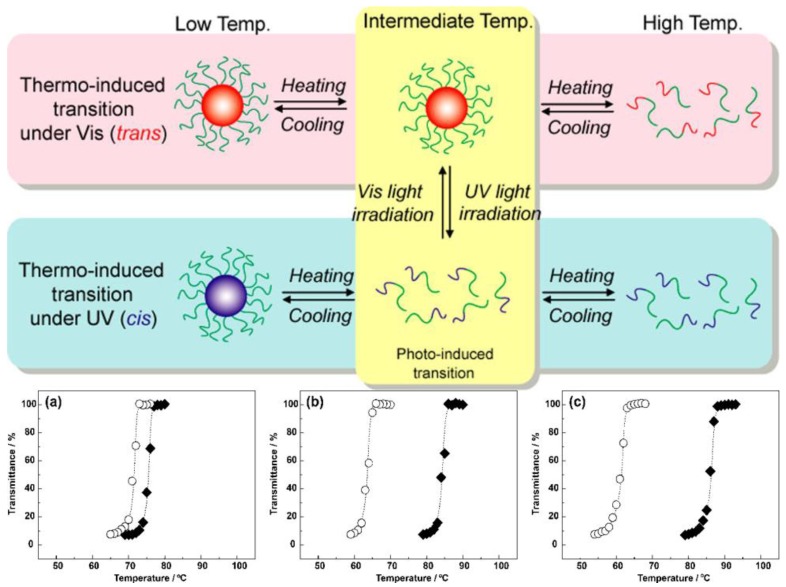
The top row highlights the characteristics of dual stimuli-responsive block copolymer micelles; below, turbidity measurements for: (**a**) p(AzoMA_1.9_-*r*-NIPAAm); (**b**) p(AzoMA_5.4_-*r*-NIPAAm); and (**c**) P(AzoMA_8.6_-*r*-NIPAAm) in [C_4_mim]PF_6_ solution are shown upon irradiation with UV light (366 nm, open circles) or in the dark (closed diamonds); the subscript denotes the respective content (mol %) of AzoMA in the random copolymers. Adapted with permission from Ref. [51]. Copyright 2012 American Chemical Society.

The first azo-dye was synthesized by Martius in 1863, and, in the following year, Griess reported the coupling reaction of related diazonium compounds [39]. Nevertheless, it took until 1937 for the reversible cis–trans-isomerization of azobenzene to be proven by Hartley [40]. After irradiation of a sample with blue light in benzene, they observed different dipole moments compared to a non-irradiated sample. This led to a different explanation for this behavior, as up to now the light response of azobenzene was ascribed to some oxidation of the compound [41]. Single molecule azobenzenes have been prepared with a great variety of substituents and exhibit excitation wavelengths starting in the far UV range [27,28] (here, the back isomerization is often mediated by thermal relaxation) to 410 nm [42] (here, the back isomerization occurs upon irradiation at 500 nm). The first polymer containing azobenzene as photo-responsive group was synthesized by Ringsdorf et al. in 1984 [43]. They first prepared the monomer 6-[4-(4-cyano-phenylazo)phenoxyhexyl acrylate and afterwards performed free radical copolymerization with the corresponding benzoate, resulting in a liquid crystalline copolymer. Some years later in 1989, Angeloni et al. compared the spectroscopic properties of main chain and side chain azobenzene polymers with the corresponding monomers and found that the substitution pattern has the largest effect on the excitation maximum [44]. This effect is also shown for block copolymers consisting of a polystyrene (PS), poly(methyl methacrylate) (PMMA), poly(β-acetyl galactose ethyl methacrylate) or poly(ethylene glycol) (PEG) block in combination with various polymeric azobenzenes [45,46,47,48,49]. In that regard, the first block copolymer containing a photo-responsive azobenzene segment was synthesized by Se et al. in 1997 using side chain modification of a polystyrene-*block*-*N,N*-dimethyl-4-vinylphenethylamine block copolymer prepared by sequential anionic polymerization with *p,p’*-Bis(chloromethyl)azobenzene [50]. In another example, the copolymerisation of methacrylate-based azobenzenes (AzoMA) with *N*-isopropyl acrylamide (NIPAAm) with various amounts of NIPAAm was shown by Ueki et al. and this significantly affected the thermal response characteristics of the resulting copolymers upon irradiation [51]. The change of the dipole moment of azobenzene was used by Concellón et al. to reversibly load aqueous block copolymer micelles (Figure 3) [52]. Here, RAFT techniques were used to prepare poly(ethylene glycol)-*block*-poly(2,6-diacylaminopyridine) (PEG-*b*-PDAP), and load these structures with *N*(1)-[12-(4-(4′-isobutyloxyphenyldiazo)phenoxy)dodecyloxy)] thymine (tAZO_i_). Afterwards, the loading could be reversibly released upon irradiation with UV-light [53].

**Figure 4:**
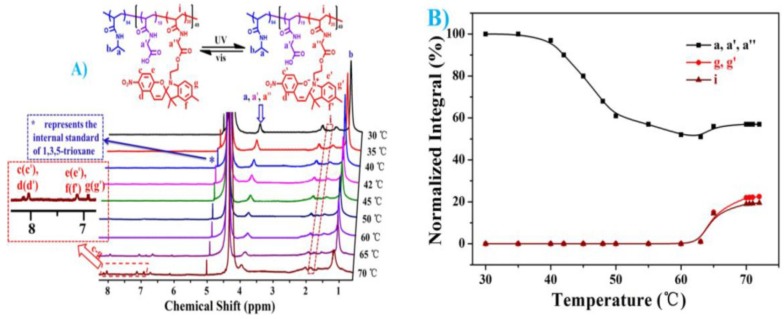
^1^H NMR spectra of 1 wt % PNIPAAm_94_-*b*-P(NAG_19_-*co*-NAGSP_30_) micelles dispersed in D_2_O at different temperatures (**A**); and the temperature-dependent signals of three typical protons of PNIPAAm_94_-*b*-P(NAG_19_-*co*-NAGSP_30_) micelles (**B**). Reproduced from Ref. [67] with permission from The Royal Society of Chemistry.

The thermally induced color change of spiropyrane-based compounds in solution has been known since the 1920s [54,55]. The origin of this color change is the light induced breakage of a bond between a tertiary carbon and a cyclic heteroatom, thereby switching between a bicyclic spiropyrane form and a zwitterionic merocyanine. Since then, a multitude of substitution patterns have been synthesized [11,56], whereas the first detailed report on the photo-response of this class of molecules was published by Fischer et al. in 1954 [57]. Benzospiropyrane molecules mainly switch to the open form upon irradiation at 365 nm and close the ring typically triggered by irradiation at 560 nm [58,59,60]. Copolymers containing a spiropyrane moiety were intensively studied by Smets in 1972, including a copolymer with MMA synthesized via free radical polymerization [61]. The first copolymer containing spiropyrane was synthesized by Krongauz et al. in 1981 by modifying benzospiropyrane with an acrylate group, followed by free radical polymerization [62]. The first reported block copolymer was of ABA-type and was prepared by De Los Santos et al. in 1999 [63]. They modified PMMA-*b*-PU-*b*-PMMA in the B segment with different spiropyrane molecules. Since then, various examples were found for amphiphilic diblock terpolymers [64,65], mainly consisting of a PEG block, and various poly(benzospiropyrane) segments with different comonomers. In general, their absorption maxima did not differ significantly from the values previously reported for the monomeric compounds [66]. The synthesis of amphiphilic block copolymers leads to many different applications from polymeric liquid crystals to photo-responsive block copolymer micelles [67,68,69,70,71]. Thereby, the formation of micelles can be triggered by addressing the spiropyrane as shown by Guragain et al. [70] or the release rate of encapsulated cargo in case of core-crosslinked micelles can be controlled as shown by Wang et al. [68]. Menon et al. [71] synthesized an amphiphilic block copolymer consisting of poly(spiropyrane methacrylate)-*block*-poly(3-*O*-4-vinylbenzoyl-d-glucopyranose), which forms 200 nm micelles in aqueous solution that can be loaded in the dark with coumarin, and release their loading upon irradiation with UV light (360 nm). They could further demonstrate that the micelles can be formed again upon irradiation with green light (560 nm). In that respect, the coupling of a thermo-responsive block to the spiropyrane-containing segment enables to control the formation of micelles also by temperature [72]. Very recently, Zhang and coworkers used RAFT polymerization to prepare P(NIPAAm)-*b*-poly(*N*-acryloylglycine) diblock copolymers, and subsequently functionalized those with benzospiropyrane ((PNIPAAm_94_-*b*-P(NAG_19_-*co*-NAGSP_30_, Figure 4). These multiple stimuli-responsive block copolymers formed spherical or worm-like micelles in water, depending on the temperature (above or below the LCST of PNIPAAm) or the irradiation with light (switching between merocyanine and spiropyrane form) [67].

Further reversible photo-responsive moieties that can be triggered in the UV region include, e.g., coumarines or anthracenes—Both being capable of undergoing cycloaddition reactions. In 1996, Liu et al. [73] synthesized polystyrene-*block*-poly(2-cinnamoylethyl methacrylate), and prepared nanofibers which were afterwards stabilized via crosslinking. The reversibility of the crosslinking process was first shown by Lendlein and coworkers in 2005, where cinnamic acid was crosslinked upon irradiation at 310 nm via (2 + 2) cycloaddition (de-crosslinking can be triggered by excitation below 260 nm) and they employed such materials in shape memory polymers [74] or reversibly cross-linkable shells in block copolymer micelles [75,76]. This can also be achieved using coumarine moieties in the side chain [77,78,79] and Zhang et al. reported on amphiphilic block copolymers of poly(ethylene oxide)-*block*-poly((*N*-methacryloxyphthalimide)-*co*-(7-(4-vinyl-benzyloxyl)-4-methylcoumarin)) and the formation of micelles thereof in aqueous media. The core can now be reversibly photo-crosslinked upon irradiation at 365 nm and this enables loading or release of various drugs such as doxorubicin [80]. Anthracene on the other hand can be crosslinked by (4 + 4) cycloadditions and Xie and coworkers used anionic ring-opening polymerization to form poly(l-lactide)-*block*-poly(ethylene glycol) functionalized with anthracene moieties in the side chain—These materials could then be used as light-responsive shape memory block copolymers [81].

#### 3.1.2. Irreversible

**Figure 5:**
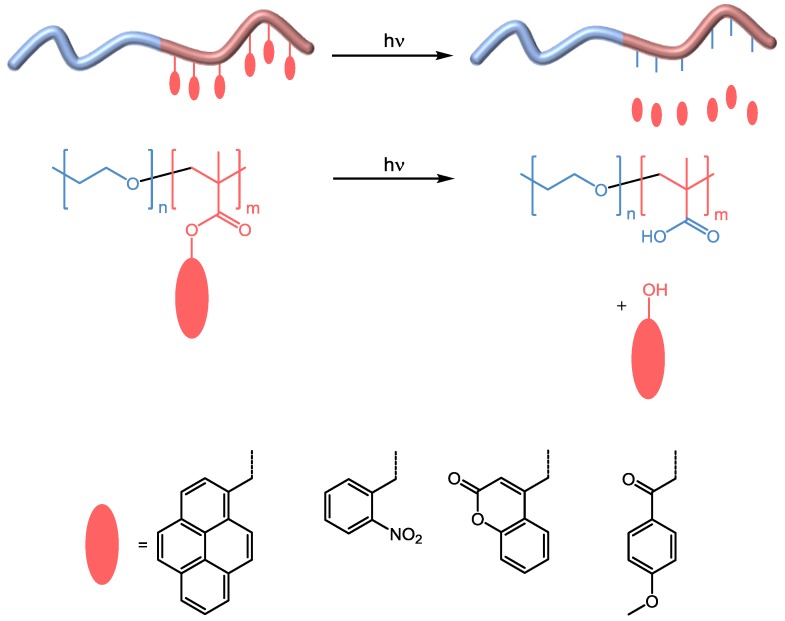
Schematic representation of UV light triggered photo-cleavage of pyrenylmethyl, *o*-nitrobenzyl, coumarinyl and *p*-methoxy-phenacyl esters within block copolymer materials.

Most irreversible photoreactions that have been reported showed response to far UV light and include photo cleavage of side groups as well as the introduction of irreversible crosslinks via photocycloadditions or photo rearrangement reactions. The former approach was essentially inspired by photo-labile protecting groups, e.g., pyrenylmethyl esters [82], *o*-nitrobenzyl esters [83], coumarinyl esters [84], and *p*-methoxy-phenacyl esters [85]—All of these have been investigated during the second half of the last century [86]. In this regard, the group of Zhao introduced two fundamental examples both presenting a block copolymer consisting of a hydrophilic PEO block and a hydrophobic and photo-responsive polymethacrylate-based segment [87,88]. The respective cleavage of either the pyrenylmethyl or *o*-nitrobenzyl groups upon UV irradiation (365 nm) shifted the hydrophilic/hydrophobic balance of the amphiphilic block copolymer through the generation of hydrophilic poly(methacrylic acid) (Figure 5). This then led to swelling or even dissociation of the micellar aggregates in aqueous media and a release of encapsulated Nile Red as model drug. Especially *o*-nitrobenzyl chromophores are discussed as promising candidates for biological applications since even stimulation with NIR light is possible (see Section 3.3). Consequently, several block copolymers bearing *o*-nitrobenzyl ester groups in the side chain in combination with blocks of PEO/PEG or POEGMA [89,90,91,92,93,94,95,96,97], polystyrene [98,99,100], poly(methyl acrylate) [101], poly(2-ethyl-2-oxazoline) [102], or polydimethylacrylamide [103] have been synthesized and investigated towards their response upon irradiation with far UV light. Exemplarily, Liu and Dong showed the photo-controlled release of the anticancer drug doxorubicin from a biodegradable polypeptide-based poly(*S*-(*o*-nitrobenzyl)-l-cysteine)-*block*-poly(ethylene oxide) block copolymer. The materials formed spherical micelles in aqueous media and exhibited a significant reduction in size after irradiation [104]. In particular, the combination of any photo-responsiveness with other stimuli, e.g., temperature, seems very favorable in that regard because inspired by natural examples a conceivable release mechanism could be initiated more effectively and thus more controlled. In that regard, dual [105,106,107,108,109,110,111,112,113,114,115,116,117,118], triple [119,120], and even quadruple responsive systems have already been presented [121]. For example, Cao et al. prepared a quadruple-responsive (light, temperature, pH and redox) poly(2-nitrobenzyl methacrylate)-*block*-poly(dimethylaminoethyl methacrylate) diblock copolymer where both blocks have been connected through a disulfide linker [122]. UV irradiation led to photo-cleavage of the *o*-nitrobenzyl groups whereas addition of the reducing agent dithiothreitol separated the blocks at the block junction, resulting in disruption of the nanoparticles (spherical structures with diameters of 80 to 140 nm) in both cases. The poly(dimethylaminoethyl methacrylate) block responded to temperature (shrinkage, diameter decreased to 20–50 nm) and to changes in pH (shrinkage to 40–60 nm at pH 9, swelling at pH 3). Furthermore, the incorporation of *o*-nitrobenzyl esters in the main chain [123,124] or as a block junction synthesized via divergent polymerization [125,126,127,128,129], convergent coupling [129,130,131,132,133,134,135,136], or even as combination of both strategies [137,138,139] represents another option to prepare photolytically cleavable amphiphilic block copolymers.

In 2009, again Zhao and coworkers demonstrated a similar concept, however they employed coumarinyl moieties instead of pyrenylmethyl and *o*-nitrobenzyl esters as photo-cleavable entities [140]. In analogy to previous studies, a PEO block was combined with a polymethacrylate-based segment containing coumarin functionalities. As another example for the controlled release of therapeutic cargo, Jin et al. reported on the release of the previously covalently bound anticancer drug 5-fluorouracil from a coumarin-functionalized polymer block under irradiation at 254 nm [141]. Quite recently, a block copolymer consisting of a PEO block and a hydrophobic segment containing both phthalimide and coumarin functional groups in the side chain was designed by Zhang et al. [80]. Irradiation with light of 365 and 254 nm wavelength led to both photo-cleavage of the phthalimide esters and simultaneous crosslinking via the coumarin groups. This approach enables the regulation of the amphiphilic imbalance and the crosslinking density of block polymer micelles simultaneously. The afore-mentioned *p*-methoxy-phenacyl esters are also the subject of more recent works by Bertrand et al. [98,142]. Thereby, the absence of a nitro group facilitates controlled polymerization procedures of the respective monomers rendering these compounds favorable if compared to the previously discussed nitrobenzyl esters. Compared to examples involving coumarin groups, photocycloadditions of cinnamic esters can be considered as a different case since most examples have been shown to be reversible. Two early studies reported about (2-cinnamoylethyl methacrylate) based block copolymers and their nanoaggregate formation in organic solvent mixtures, followed by (at least according to these descriptions) irreversible photo-crosslinking experiments [143]. In 2012 the same group demonstrated a dual light-responsive triblock terpolymer consisting of a *o*-nitrobenzyl ester block junction as well as a crosslinkable poly(2-cinnamoylethyl methacrylate) (PCEMA) block [144,145]. Photolysis of THF/water mixtures (80% water, particle sizes: 20–50 nm in diameter) led to both cleavage of the hydrophilic PEO block and a precipitation of the now core-crosslinked nanoparticles. Another example by Yang et al. presented the use of the cinnamic acid (2 + 2) cycloadduct, truxillic acid, as a block junction and demonstrated its use for the photo cleavage of a poly(ethylene glycol)-*block*-poly(acrylate) diblock copolymers [146]. In that context, light induced rearrangement reactions open up another strategy to induce a sudden shift in the hydrophilic/hydrophobic balance of block copolymers. Among others, two different examples, i.e., the Wolff rearrangement of diazonaphtoquinones [147] and the photo-Claisen rearrangement of allylphenyl ethers [148], have been applied to the field of block copolymers. Other irreversible UV light induced photo-responsive block copolymers include the application of photo-decomposable polyurethanes [149,150] and photoacid generators [151]. In the latter case, a PMMA block was combined via sequential RAFT polymerization with a segment containing photoacid-generating sulfonium groups. The self-assembly in the bulk and the lithographic properties in the course of the photochemical reaction were investigated.

### 3.2. Vis (400–600 nm)

#### 3.2.1. Reversible

**Figure 6:**
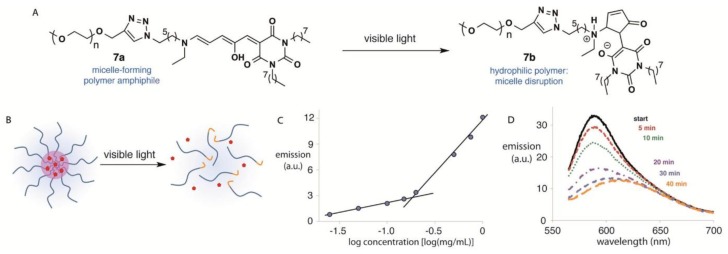
(**A**) Photo-switching of a DASA-functionalized polymeric amphiphile; (**B**) schematic of micelle formation and hydrophobic cargo encapsulation by a photo-responsive amphiphile and micelle disruption and cargo release upon irradiation with visible light; (**C**) fluorescence intensity (emission at 588 nm) vs. log concentration (mg/mL) of the polymeric amphiphile; and (**D**) fluorescence emission spectra of Nile Red in 0.50 mg/mL of the polymeric amphiphile in water at various times of irradiation. Reprinted with permission from Ref. [152]. Copyright 2014 American Chemical Society.

The Donor–Acceptor Stenhouse Adduct (DASA) is relatively new to the class of photochromic molecules (Figure 6) [152], whereas the underlying Stenhouse adduct itself is already known since 1850 [153]. Helmy et al. first functionalized a PEG segment with this group and afterwards showed that the resulting materials reversibly respond to irradiation with visible light (570 nm) in toluene, which can also be used for cargo release upon incorporation into micellar systems (Figure 6). DASA was first used in polymeric form by Balamurugan in 2016 by copolymerizing glycidyl methacrylate (GMA) and dimethylacrylamide (DMA) via RAFT polymerization to yield poly(glycidyl methacrylate-*co*-dimethylacrylamide) [P(GMA-*co*-DMA)] [154]. This was then modified with DASA and showed excellent photochromic performance upon irradiation with a crystal clear halogen lamp. It was also shown by Sinawang et al. that it is possible to introduce DASA into the side chain by post polymerization functionalization of poly(styrene-*co*-4-vinylbenzyl chloride) copolymers [155].

**Figure 7:**
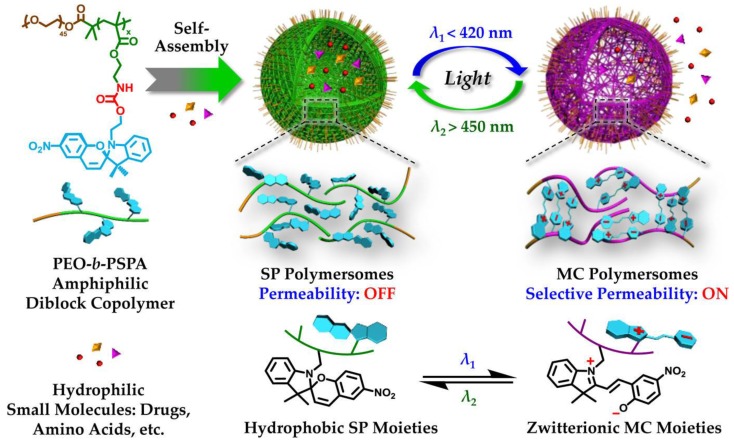
Amphiphilic PEO-*b*-PSPA diblock copolymers self-assemble into polymersomes with hydrophobic bilayers containing carbamate-based hydrogen-bonding motifs; the spiropyran moieties within the polymersome bilayers undergo reversible photo-triggered isomerization between hydrophobic spiropyran (SP, λ_2_ > 450 nm) and zwitterionic merocyanine (MC, λ_1_ < 420 nm) states. Reprinted with permission from Ref. [68]. Copyright 2015 American Chemical Society.

Photo-responsive molecules that can be addressed by irradiation with visible light are of great interest, therefore a redshift by varying the substitution pattern for different chromophores has been discussed in a review by Bleger et al. [156], although only few examples have been incorporated into block copolymers so far. By modification of the linkage from the benzospiropyrane side chain functionality to the block copolymer backbone it was possible for Wang [68] and coworkers to synthesize poly(ethylene glycol)-*block*-poly(spiropyranemethacrylate) (PEG-*b-*PSPMA) diblock copolymers. These amphiphilic materials self-assembled in aqueous media into vesicles, which could be loaded with doxorubicin, gold nanoparticles, or various fluorescence markers. The microstructures of both spiropyrane and merocyanine polymersomes are synergistically stabilized due to hydrophobic interactions, hydrogen bonding, π−π stacking, and electrostatic (zwitterionic) interactions, with the latter two types being exclusively found for MC polymersomes. Moreover, the reversible photo-triggered SP/MC polymersome transition is accompanied by changes in membrane permeability—Thereby shifting from being non-permeable (450 nm) to selectively permeable (420 nm) towards non-charged, charged, and zwitterionic small molecule species below a critical molar mass (Figure 7) [68].

Although not focusing on solution structures, it was further shown by Yu et al. in 2006 that a linearly polarized laser beam (488 nm) can be used to control the self-assembly of nanocylinders from an amphiphilic liquid-crystalline diblock copolymer consisting of flexible poly(ethylene oxide) as hydrophilic block and a poly(methacrylate) containing an azobenzene moiety in the side chain as a hydrophobic liquid-crystalline segment (PEO_114_-*b*-PMAAz_60_) [157]. Upon irradiation at 488 nm, these PEO cylinders could be reoriented in perpendicular direction.

#### 3.2.2. Irreversible

Only few examples have been developed where photo-responsive block copolymers have been activated within the visible light regime. Besides, a classification concerning the actually used chromophores similar to that seen for the above-mentioned systems is not equally straightforward. However, two different general strategies can be identified so far. First, photo-cleavage mechanisms have to be discussed and, in that regard, Sun et al. reported the red-light mediated (520 nm) cleavage from block copolymers consisting of a PEG block and a 6-(4-cyanophenoxy) hexyl methacrylate block that in certain amounts coordinates ruthenium complexes which in turn are potential anticancer metallodrugs [158]. Another example by Zhou and coworkers presented similarly an ABA triblock copolymer with water soluble PEG as A segment and hydrophobic polyurethane having a Pt(IV) prodrug linked to the backbone as the middle block B [159]. Under irradiation with UV or visible light a conversion to Pt(II) occurred which in vivo (demonstrated with BALB/c nude mice) enabled binding to DNA, finally resulting in cell death.

**Figure 8:**
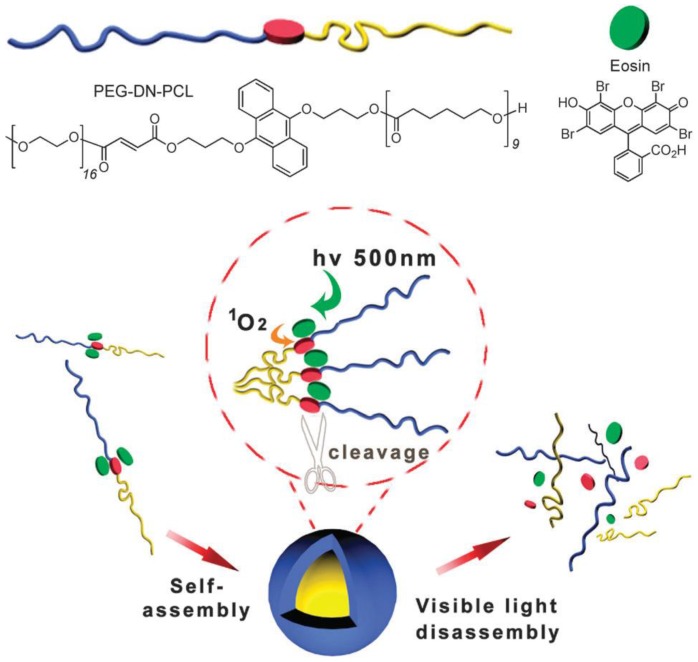
Schematic representation of a poly(ethylene glycol)-*block*-poly(caprolactone) amphiphilic block copolymer featuring a dialkoxyanthracene block junction, its self-assembly and visible light-triggered disassembly via photo-cleavage of the block junction. Reproduced from Ref. [160] with permission from the Royal Society of Chemistry.

The second approach represents in principle also a cleavage reaction, although here the process is mediated by the presence of singlet oxygen. For instance, by using an eosin sensitizer that responds to visible light sources and produces singlet oxygen, dialkoxyanthracenes can be converted to 9,10-anthraquinones by cleavage of the alkoxy moieties. This principle was demonstrated for a poly(ethylene glycol)-*block*-poly(caprolactone) PEG-*b*-PCL amphiphilic block copolymer consisting of a dialkoxyanthracene block junction in 2012 (Figure 8) [160]. Alternatively, porphyrin derivatives can be used as sensitizers for a subsequent cleavage like shown for an ABA-type triblock copolymer with a singlet oxygen sensitive diselenide-containing polyurethane B middle block surrounded by PEG segments [161]. Saravanakuma et al. presented a similar approach for a poly(ethylene glycol)-*block*-poly(caprolactone) diblock copolymer with a cleavable vinyldithioether block junction [162]. In both cases, the visible light mediated release of doxorubicin as a hydrophobic model drug was tested. Quite recently, also amphiphilic diblock copolymers with one block featuring a diselenide linkage in the side-chain were reported [163]. Visible light exposure induced diselenide exchange and, thereby, crosslinking of these drug-loaded nanocarriers which then in turn are capable of undergoing redox-responsive release in close vicinity to a tumor.

### 3.3. Near IR (700–1000 nm)

Near infra-red (NIR) light-responsive block copolymers are becoming more and more popular since they represent a promising opportunity to overcome issues that are combined especially with UV light irradiation (poor tissue penetration and toxic side effects) [2,3]. Recently, a review article focusing on photo-responsive materials for NIR stimulation has been published by Cho et al. [164]. Besides photo-induced heating strategies, NIR-triggered photoreactions have also been outlined and discussed. The respective examples and some recent work will be discussed in the following section. Here, we decided to focus on irreversible approaches because reversible NIR-responsive block copolymers have not been reported yet. Instead, the general concept using nanoparticles capable of light upconversion addressing photo-responsive block copolymers (mainly after micellization) will shortly be presented as well.

#### 3.3.1. Irreversible

**Figure 9:**
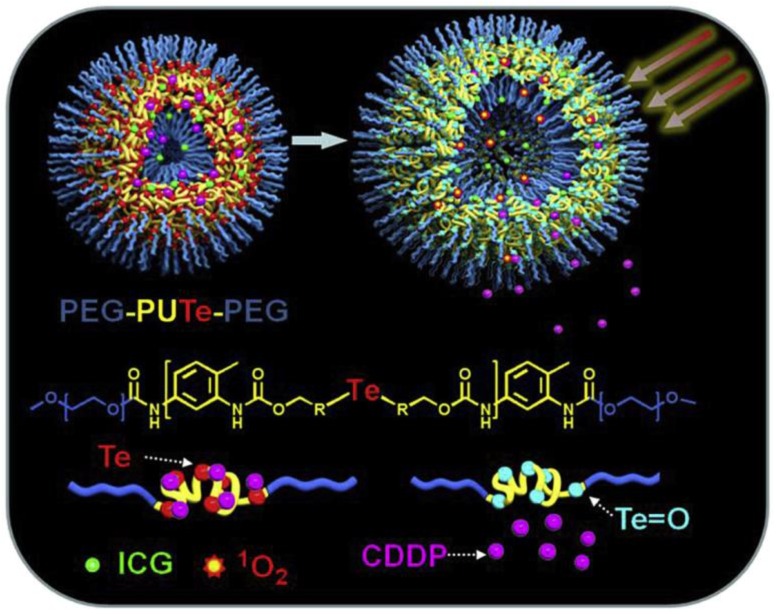
NIR light-triggered release of cisplatin (here abbreviated with CDDP) and indocyanine green (ICG) from an amphiphilic block copolymer micelle; stimulation of ICG leads to the formation of ^1^O_2_ and the oxidation of tellurium, thereby drastically weakening the tellurium-cisplatin coordination. Reprinted from Ref. [172] with permission from Elsevier.

Most examples for irreversible photoreactions in the NIR range are closely connected to the above mentioned studies for far UV light responsive block copolymers since *o*-nitrobenzyl and coumarinyl esters show also photo-cleavage in the NIR window in terms of two-photon absorption occurring [165,166]. This has been already discussed in the early reports by the group of Zhao where they investigated the stimulation via NIR irradiation [88,140]. Cao et al. presented the preparation of polysaccharide-based *N*-succinyl-*N′*-4-(2-nitrobenzyloxy)-succinyl-chitosan amphiphilic block copolymer micelles containing *o*-nitrobenzyl ester groups and further demonstrated the conjugation with a tumor targeting ligand and an encapsulated antitumor drug [167,168]. It is noteworthy that, in addition to the fact that potentially toxic side-products (nitrosobenzaldehyde) are formed, the NIR-triggered reaction of *o*-nitrobenzyl esters usually exhibits rather long reaction times [169]. One proposed solution that was presented by Zhao and coworkers is the application of more efficient NIR two-photon-absorbing chromophores [170]. Accordingly, they synthesized biocompatible poly(ethylene oxide)-*block*-poly(l-glutamic acid) bearing 6-bromo-7-hydroxycoumarin-4-ylmethyl groups and showed the release of two different drug molecules upon irradiation with 794 nm. Another example by Ji et al. showed a block copolymer containing a coumarin functionalized block and another block of poly(hydroxyethylacrylate) which was successfully conjugated with folic acid as a selective cancer target compound [171]. Through hydrophobic interactions, the block copolymer was adsorbed onto hollow silica nanoparticles modified with hydrophobic octadecyl chains. The resulting nanocontainers were pre-loaded with doxorubicin and subsequently controlled NIR light triggered drug release was performed. Very recently, the same group which reported in 2013 on singlet oxygen mediated cleavage of a diselenide-bridged polyurethane middle block surrounded by PEG segments triggered by visible light (mentioned in Section 3.2.2) presented a similar system but containing tellurium as responsive junction point [163]. Here, the tellurium is coordinating cisplatin and a co-loaded FDA approved NIR dye for photodynamic therapy (indocyanine green (ICG)) which is acting as a sensitizer to generate singlet oxygen. Oxidation of tellurium led to both the release of cisplatin and the ICG in turn which in sum increased the anti-tumor efficacy when compared with the treatment of cisplatin alone (Figure 9).

#### 3.3.2. Upconversion

**Figure 10:**
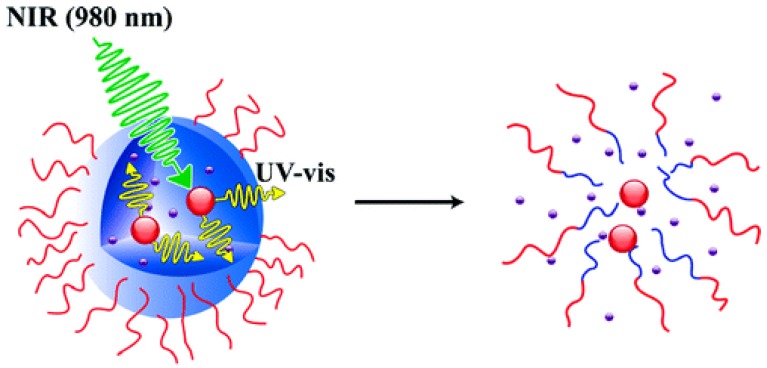
Schematic representation of NIR to UV light conversion via UCNP inside a block copolymer micelle and resulting release of encapsulated guest molecules. Reproduced with permission from Ref. [175]. Copyright 2011 American Chemical Society.

Upconverting nanoparticles (UPNP) efficiently absorb NIR light and convert it to lower wavelengths but, even more important, they can be used to assist photochemistry in the UV/Vis range [173]. In that regard, Carling et al. were the first who demonstrated a remotely controlled photoswitching of dithienylethene compounds by using UCNPs of NaYF_4_:TmYb and NaYF_4_:ErYb which converted 980 nm NIR light to trigger the UV/Vis responsive process [174]. In 2011, the same authors in cooperation with Zhao and coworkers presented a model system consisting of NaYF_4_:TmYb upconverting nanoparticles inside poly(ethylene oxide)-*block*-poly(4,5-dimethoxy-2-nitrobenzyl methacrylate) block copolymer micelles (Figure 10) [175]. The desired internal UV light source was used to cleave off the *o*-nitrobenzyl functions leading to dissociation of the micelles and in turn to a release of co-loaded Nile red which was confirmed by fluorescence emission measurements.

Similarly, a PEO-*b*-P(NIPAM-*co*-NBA) adsorbed onto an UCNP was presented in 2014 and again used for Nile red release experiments [176]. In 2017, a PEG-*b*-PS block copolymer with a block junction having both an *o*-nitrobenzyl moiety and an azobenzene group incorporated was photo-triggered in the presence of UCNPs resulting in disruption of the nanoaggregates [177]. Thus far, no examples for spiropyrane containing block copolymers in combination with UCNP have been demonstrated. For direct modification of these photo-responsive chromophores via UCNPs, the reader is referred to a recent review by Wu and Butt [178]. However, statistical copolymers having spiropyranes incorporated have been already attached to UCNP by Chen and coworkers [179]. These nanoparticles were used to polymerize poly(NIPAAm-*co*-spiropyrane methacrylate) and reversible switching of spiropyrane to the merocyanine form was achieved upon irradiation with light at 980 nm [180]. In another example, lanthanide-based UCNPs co-doped with Yb^3+^ and Tm^3+^ were encapsulated within mesoporous silica and coated with a methacrylate/methacrylamide terpolymer consisting of spiropyrane and PEG grafted groups as well as side-chain conjugated folic acid functions used as receptors for tumor cell targeting [181]. Beforehand, doxorubicin could be loaded into the mesoporous silica layer and the respective release studies were carried out, both in vitro and in vivo.

## 4. Conclusions and Outlook

Clearly, the field of photo-responsive block copolymers is still evolving, especially when it comes to materials or examples where irradiation has to be carried out at wavelengths distinctly higher than the far UV regime. However, one thing that becomes evident when comparing different approaches reported so far is that it can be difficult to directly compare photo-response of different reports, as often entirely different irradiation setups are used. In other words, quantitative evaluation of photo-response in nanostructured materials alongside with issues such as complete/incomplete reversibility, determination of the amount of unaffected chromophores, or long-time photo-stability assessment is sometimes difficult to judge. This even translates into entirely different classifications of the term “photo-responsive” as well as a broad variety of light sources being used—Sometimes also without determination of the overall light intensity.

Another aspect that is not always considered is whether continuous or repetitive irradiation also leads to photo-damage of the underlying polymer/block copolymer backbone, especially if UV light is used. Nevertheless, many very interesting and promising studies have been reported during the last decade and, although there is a certain variety of applications for such materials, the main research direction with regard to photo-responsive block copolymers in our opinion is towards improved control over spatial and temporal release of encapsulated cargo. Thereby, many examples have been reported where amphiphilic block copolymers containing one segment with covalently or non-covalently attached chromophores self-assembled into micelles within aqueous media and irradiation with light of a suitable wavelength then leads to (burst) release or degradation. In the latter case, irradiation is often accompanied by a sudden increase in solvent quality for the core of such aggregates, leading to swelling and, thus, permeability for guest molecules or even to complete dissolution if the resulting segment then is sufficiently hydrophilic. Nevertheless, most studies still deal with model drugs such as Doxorubicin or even Nile Red and the next step forward, possibly involving methods of upconversion to trigger release, can be anticipated within the next few years.
